# Hyperhomocysteinemia and MTHFR Polymorphisms as Antenatal Risk Factors of White Matter Abnormalities in Two Cohorts of Late Preterm and Full Term Newborns

**DOI:** 10.1155/2015/543134

**Published:** 2015-02-10

**Authors:** Lucia M. Marseglia, Antonio Nicotera, Vincenzo Salpietro, Elisa Giaimo, Giovanna Cardile, Maria Bonsignore, Angela Alibrandi, Daniela Caccamo, Sara Manti, Gabriella D'Angelo, Carmelo Mamì, Gabriella Di Rosa

**Affiliations:** ^1^Department of Pediatric, Gynecological, Microbiological and Biomedical Sciences, Unit of Neonatal Intensive Care, University of Messina, Messina, Italy; ^2^Department of Pediatric, Gynecological, Microbiological and Biomedical Sciences, Unit of Child Neurology and Psychiatry, University of Messina, Messina, Italy; ^3^Department of Pediatric, Gynecological, Microbiological and Biomedical Sciences, Unit of Pediatric Genetics and Immunology, University of Messina, Messina, Italy; ^4^Department of Economical, Business and Environmental Sciences and Quantitative Methods, University of Messina, Messina, Italy; ^5^Department of Biomedical Sciences and Morpho-Functional Imaging, University of Messina, Messina, Italy; ^6^Department of Pediatric, Gynecological, Microbiological and Biomedical Sciences, Unit of Neonatology, University of Messina, Messina, Italy

## Abstract

Higher total homocysteine (tHcy) levels, and C677T and A1298C methylenetetrahydrofolate (MTHFR) polymorphisms, have been reported in preterm or full term newborns with neonatal encephalopathy following perinatal hypoxic-ischemic insult. This study investigated the causal role of tHcy and MTHFR polymorphisms together with other acquired risk factors on the occurrence of brain white matter abnormalities (WMA) detected by cranial ultrasound scans (cUS) in a population of late preterm and full term infants. A total of 171 newborns (81 M, 47.4%), 45 (26.3%) born <37 wks, and 126 (73.7%) born ≥37 wks were recruited in the study. cUS detected predominant WMA pattern in 36/171 newborns (21.1%) mainly characterized by abnormal periventricular white matter signal and mild-to-moderate periventricular white matter volume loss with ventricular dilatation (6/36, 16.6%). WMA resulted in being depending on tHcy levels (*P* < 0.014), lower GA (*P* < 0.000), lower Apgar score at 1 minutes (*P* < 0.000) and 5 minutes (*P* < 0.000), and 1298AC and 677CT/1298AC genotypes (*P* < 0.000 and *P* < 0.000). In conclusion, both acquired and genetic predisposing antenatal factors were significantly associated with adverse neonatal outcome and WMA. The role of A1298C polymorphism may be taken into account for prenatal assessment and treatment counseling.

## 1. Introduction

Homocysteine (Hcy) is a sulfur-containing amino acid involved in methionine metabolism. Either genetic or environmental factors would influence total Hcy (tHcy) levels [1]. Cofactors deficiency or genetic polymorphisms of the key catabolic enzyme and methylenetetrahydrofolate reductase (MTHFR) may be the cause of increased levels of plasma homocysteine [1]. A direct correlation between higher tHcy levels and C677T and A1298C MTHFR polymorphisms has been reported in some populations of preterm or full term newborns with neonatal encephalopathy (NE) following perinatal hypoxic-ischemic (HI) insult [2]. However, the specific role of hyperhomocysteinemia and MTHFR polymorphisms in neonatal brain maturation and HI damage has not been unraveled. Mild hyperhomocysteinemia and inflammation have been associated with the development of several cerebrovascular diseases [3]. Mechanisms as excitotoxicity and activation of oxidative stress were related to the toxic effects of homocysteine [3]. Two definite patterns of brain injury were associated with NE following HI insult, predominantly involving white matter/watershed (WM/WS), or the basal ganglia/thalamus (BGT) regions [2, 4]. The association between WM/WS pattern, tHcy levels, and either heterozygous or homozygous MTHFR C677T variants has been reported [2]. Nevertheless, beyond the mostly definite pattern of white matter injury such as periventricular leukomalacia (PVL), several qualitative neonatal white matter abnormalities (WMA) may account for different degrees of neurobehavioral impairment on long-term observation, mainly in very preterm [5, 6] but also in full term infants [4]. Both environmental events and predisposing genetic vulnerability were hypothesized as causative factors of WMA [7]. The aim of our study was to evaluate the role of tHcy and MTHFR polymorphisms together with other acquired risk factors on the occurrence of WMA detected by cranial ultrasound scans (cUS) in late preterm and full term infants.

## 2. Subjects and Methods

### 2.1. Clinical Data

Newborn infants were consecutively recruited at the Intensive Care Unit and Neonatology Unit of University Hospital of Messina between February 2011 and February 2013. Approval from the Local Ethical Committee was obtained for this study. Medical records were reviewed systematically for details concerning antenatal, perinatal, and neonatal course. Demographic and clinical data included sex, type of pregnancy (pathologic versus physiologic; singleton versus twin), drugs taken during pregnancy (steroids, tocolytics, progesterone, cardioaspirin or low-molecular-weight heparin (LMWH)), mode of delivery (emergency cesarean section), preeclampsia, premature rupture of membranes (PROM), birth weight (BW; expressed in percentiles), gestational age (GA), Apgar score (1 and 5 min), patent foramen ovale (PFO), perinatal adversities (bradycardia, altered flussimetry, seizures, infections, hypoglycemia, and multiorgan failure). Clinically recognizable neonatal encephalopathy after presumed HI was defined by abnormal tone pattern, feeding difficulties, altered alertness, or seizures, and at least one of the following criteria: (1) fetal distress at delivery, (2) requirement of resuscitation at birth, (3) arterial cord blood pH <7.1, (4) 5-minute Apgar score <7, or (5) multiorgan failure [8]. Exclusion criteria included patients with GA below 32 wks, suspected or confirmed congenital brain malformations, inborn errors of metabolism, neuromuscular diseases, or trisomies.

### 2.2. Biochemical and Genetic Analyses

For measurement of tHcy levels in whole blood, samples were collected with 20 *μ*L of sodium heparinized capillaries (Bio-Rad Laboratories, Munich, Germany) within 1 week from birth. Then, blood samples were hemolyzed with 0.2 mL hemolysis reagent (aqueous solution of EDTA and a nonionic detergent; Bio-Rad Laboratories), resulting in an immediate inhibition of Hcy-generating and -converting enzymes and a stabilization of the tHcy concentration for up to 2 days. The hemolyzed blood samples were stored at +4°C for a maximum of 2 days until analysis. Measurements of plasma tHcy concentrations were performed by HPLC with kit dedicated (Bio-Rad Laboratories), according to manufacturer's instructions.

### 2.3. Genotyping of MTHFR Polymorphisms

The assessment of C677T and A1298C MTHFR genotypes was carried out on genomic DNA extracted from dried blood spots (DBS) samples. Written informed consent was obtained from patients' parents. DBS were obtained by heel prick with a sterile lancet, after applying filter paper to draining blood. Disks of 3 mm were cut out of the sampling paper and placed into 1.5 mL Eppendorf minitubes. Then, 500 *μ*L sterile water was added, and the minitubes were vortexed 3 times during 5 seconds. The water was pipetted off. After adding 200 *μ*L 10% Chelex-100 solution, the minitubes were placed in a water bath at 95°C for 30 minutes. Finally, these ready for use DNA solutions were pipetted into new minitubes. After DNA spectrophotometric quantification, genotyping for the C677T and A1298C polymorphisms was carried out by allelic discrimination technique in a 7500 real-time PCR instrument (Applied Biosystems), using Pre-Designed TaqMan_ SNP Genotyping Assays (Applied Biosystems; assay ID: C_1202883_20 and C_850486_20, resp.), according to manufacturer's instructions. The frequency of C677T and A1298C MTHFR polymorphisms was compared between premature (GA < 37) versus born-at-term newborns (GA ≥ 37).

### 2.4. Neuroimaging Studies

Cerebral ultrasound was performed using an Esaote AUS with a 7.5 MHz sector probe transducer to all newborns by the same experienced neonatologist. Serial cUSs were performed from 1 to 3 days of life and 44 postmenstrual age. Neonatal white matter abnormalities (WMA) were defined according to the presence of one of the following patterns: (1) white matter signal abnormalities, (2) periventricular white matter volume loss, (3) the presence of any cystic abnormalities, (4) ventricular dilatation, and (5) thinning of the corpus callosum [5].

### 2.5. Statistical Analyses

Numerical data were expressed as median and range and the categorical variables as numbers and percentages. Because numerical variables did not present normal distribution as verified by* Kolmogorov Smirnov test* and most of the measured variables were dichotomous or categorically ordered, nonparametric approach was used [9].

The* Spearman correlation test* was applied in order to assess the existence of interdependence between tHcy levels and other numerical parameters [9].

In order to assess the association between categorical variables (such as preterm versus full term newborns and MTHFR genotypes) we performed* Chi-Square test*.

*Odds ratio* and related confidence interval at 95% level were estimated in order to quantify the risk of premature birth for heterozygous MTHFR forms [9].

*Mann-Whitney test* was performed in order to compare tHcy levels between the groups of preterm versus full term infants and between WMA versus normal cUS scans.* Kruskall-Wallis test* was performed to compare tHcy levels among newborns with different Apgar scorings defined as normal (10–7), moderately impaired (6–4), and severely impaired (below 4) [9].

Finally, some* logistic regression models* [8] were estimated to verify the possible dependence of the WMA (in terms of Yes or NO) from GA, altered flussimetry, preeclampsia, PROM, bradycardia, tHcy levels, and MTHFR genotype. The same model was applied in order to assess the dependence of intraventricular hemorrhage (IVH) from the same potential explicative variables.* Linear regression models* were estimated in order to assess the dependence of numerical variables, such as BW, GA, and Apgar scores at 1 min and 5 min from tHcy levels. The same model was applied to ascertain the dependence of tHcy levels from drugs assumption during pregnancy (steroid, tocolytics, progestins, and cardioaspirin).

Statistical analyses were performed using SPSS 11.0 for Windows package. *P* < 0.05, two-tailed, was considered to be statistically significant.

## 3. Results

### 3.1. Demographic and Clinical Features of the Sample

Summarized demographic, clinical, biochemical, and molecular features are shown in Table 1. A total of 171 newborns (81 M, 47.4%), 45 (26.3%) born <37 wks, and 126 (73.7%) born ≥37 wks were recruited. Pregnancy was physiologic in 104/171 (60.8%), pathologic in 67/171 (39.2%), single in 157/171 (91.8%), and multiple in 14/171 (8.2%). Drugs' intake during gestation included steroid in 33/171 (19.3%), tocolytics in 23/171 (13.5%), progesterone in 47/171 (27.5%), and cardioaspirin in 4/171 (2.3%). Antenatal complications included preeclampsia in 5/171 (2.9%) and PROM in 3/171 (1.8%). Perinatal complications included bradycardia in 18/171 (10.5%), altered flussimetry in 6/171 (3.5%), and meconium-stained liquor in 3/171 (1.7%). Neonatal data included the following: mean Apgar score (1 min) was 8.25, SD ± 1.7 (median 9; range 0–10), mean Apgar score (5 min) was 9.35, SD ± 1.1 (median 10; range 3–10), mean BW percentile was 48.95, SD ± 28.1 (median 50; range 2–90), and mean GA was 37.2 SD ± 2.6 (median 38; range 32–41). Perinatal infections were diagnosed in 10/171 (5.8%) of patients. Neonatal seizures occurred in 5/171 (2.9%) of patients. Hypoglycemia < 2.0 mmol/L or multiorgan failure was evidenced in none of the patients.

### 3.2. Neuroimaging Findings

cUS showed that 36/171 subjects (21.1%) presented predominant WMA pattern mainly characterized by abnormal periventricular white matter signal and mild-to-moderate periventricular white matter volume loss with ventricular dilatation (6/36, 16.6%). Moreover, 2/171 (1.1%) showed BGT abnormalities, 13/171 (7.6%) had intraventricular hemorrhage (IVH grade I–III), and 122/171 (71.3%) showed unremarkable cUS scans. Ventricle-peritoneal shunt catheter was placed in 6 of the 13 patients (46.1%) with IVH who developed hydrocephalus.

### 3.3. Biochemical Findings

No significant difference arose between mean tHcy values in premature infants (10.55 (SD ± 4.29) mM/L, median 10.10 mM/L (range 5–26)) and full term infants (9.74 (SD ± 3.98) mM/L, and median 9.55 mM/L (range 1.7–33)). Moreover, although, mean tHcy levels were higher in subjects with WMA pattern compared to those without cUS abnormalities, the difference only approached statistical significance (*P* < 0.081). Among the 171 subjects, 86 (50.2%) showed tHcy values above the reference ranges for children age as for our laboratory (9 to 9.5 mM/L) (Table 1).

### 3.4. Genetic Studies

Seventy-seven out of 171 subjects performed genetic analyses. The variants were distributed as CT (57.1%) and TT (10.4%) for C677T, AC (33.8%) and CC (2.6%) for A1298C. The genotype 677T/1298C was detected in 22 (28.6%) subjects.

According to GA, MTHFR C677T polymorphisms were detected in 39/77 (50.6%) preterm and in 38/77 (49.4%) full term newborns, whereas MTHFR A1298C polymorphisms were evidenced in 21/39 (53.8%) preterms and in 7/38 (18.4%) full terms. Within the group of preterm infants, 25/39 (64.1%) bore 677CT genotype, 4/39 (10.2%) bore 677TT genotype, 20/39 (51.2%) bore 1298AC genotype, and 1/39 (2.5%) bore 1298CC genotype. Within the group of full term infants, 19/38 (50%) bore 677CT genotype, 4/38 (10.5%) bore 677TT genotype, 6/38 (15.7%) bore 1298AC genotype, and 1/38 (2.6%) bore 1298CC genotype (Table 1). Each genotype was in* Hardy-Weinberg equilibrium*.

### 3.5. Statistical Results

Significant correlations emerged between tHcy and PROM (*P* < 0.013), between tHcy and PFO (*P* < 0.042), and between tHcy levels and genotypes 1298AC (*P* < 0.000) and 677CT/1298AC (*P* < 0.000). In contrast, no significant correlations emerged between tHcy and sex, pregnancy type, steroids, tocolytics, progestins and cardioaspirin therapies, emergency cesarean section, preeclampsia, bradycardia, flussimetry, BW, GA, Apgar score at 1 and 5 mi, IVH and neonatal seizures. A strongly significant risk of premature birth was detected in association with MTHFR A1298C polymorphisms (OR = 5.61; 95% CI = 1.91–16.44). In contrast, a moderate risk emerged between the MTHFR C677T polymorphisms and prematurity (OR = 1.78 for CT; 95% CI = 0.71–4.44). Neonatal WMA pattern significantly covariated with MTHFR 677CT genotype (*P* < 0.041), 1298AC genotype (*P* < 0.000), and 677CT/1298AC (*P* < 0.000). Regression analysis showed that higher tHcy levels significantly influenced Apgar score at 5 min (*P* < 0.002) and occurrence of PROM (*P* < 0.007). Moreover, WMA resulted to be dependent on tHcy levels (*P* < 0.014), lower GA (*P* < 0.000), lower Apgar score at 1 minutes (*P* < 0.000) and 5 minutes (*P* < 0.000), 1298AC, and 677CT/1298AC genotypes (*P* < 0.000 and *P* < 0.000). Statistical results are shown in Tables 2 [Table tab3]–4.

## 4. Discussion

The predisposing role of high tHcy levels and MTHFR polymorphisms on the occurrence of WMA has been strongly evidenced in our study, both in full-term and late preterm newborns. However, WMA was also significantly associated with acquired factors as GA and perinatal status, measured by Apgar scores. Several studies investigated on the origin and time of the neonatal brain lesions both in preterm and full term infants with NE [4, 10–13]. The role of intrapartum asphyxia in etiopathogenesis of NE has been debated and major neurological sequelae may not be evidenced in children who experienced fetal distress and perinatal asphyxia [14–16]. Mercuri et al. showed how 28% of newborns presenting with NE with Apgar scores <3 had normal brain MRI scans or minor WMA, whereas 95% of the infants with Apgar scores >7 and abnormal clinical assessment at 48 hours after birth had abnormal scans [11]. Cowan et al. reported that the pattern of brain injury depends on the duration and severity of the HI insult [10]. However, although the authors found that more than 90% of full term newborns with NE had a history of perinatal acquired insults, they could not exclude an additional role of other antenatal predisposing factors that make children more susceptible to the stressors during labour and delivery [10]. Moreover, the majority of patients with evidence of NE showed predominant localization of brain lesions in BGT and cortex regions, whereas the white matter regions appeared to be relatively spared within this group of children [10]. Notably, some authors reported that white matter damage is more seemingly related to long-standing antenatal risk factors interfering with brain development [4], and the state of brain maturation is an important prerequisite for this pattern of brain injury in full term newborns with NE [4, 17]. The great contribution of lower GA at birth supported the hypothesis that development of WMA was critically related to maturation state of the brain [17]. The pathogenesis of WMA has been attributed to the intrinsic vulnerability of two cell types of the developing brain: preoligodendrocytes (pre-OLs) and subplate neurons [18, 19]. These cells result in being involved in critical developmental events between 24 and 40 weeks of GA [18], possibly disrupted by premature birth or intrauterine exposure to chronic hypoxic or inflammatory events [17, 20, 21]. Because of the rapidity and complexity of these changes, immature neural cells are deemed to be particularly vulnerable to various exogenous and endogenous insults, such as ischemia, inflammation, excitotoxicity, and free-radicals attack [18]. Determinants of the relative role of each scenario could be some factors such as GA, the timing and nature of the insult, additional risk factors such as disturbed nutritional state, drugs exposure, and many still-to-be-defined environmental or genetic parameters [18]. Within this complex context, Hcy might display its action by mediating oxidative stress activation, neurotoxicity, endothelial damage, or impaired blood coagulation [22]. A toxic role of Hcy on brain cortical and subcortical structures has also been reported to be mediated by the inhibition of Na+/K+ ATPase. Several studies showed that chronic administration of Hcy to experimental animals induced a significant reduction of the activity of Na+/K+ ATPase in cerebral cortex and hippocampus [23]. The critical role of this membrane protein on maintenance of ions homeostasis and subsequent neuronal excitability as well as transport and neurotransmitters uptake is well known [23]. Nevertheless, the activity of Na+/K+ ATPase is known to be decreased by hypoxic-ischemic insults that, in central myelinated axons, lead to the accumulation of axoplasmic Na through noninactivating Na channels, which, together with membrane depolarization, promotes reverse Na-Ca exchange and axonal Ca overload. Excessive accumulation of cytosolic Ca in turn activates various Ca-dependent enzymes resulting in irreversible injury [24]. Excitotoxic mechanisms induced by hypoxic-ischemic conditions also play an important role: glutamate released by reversal of Na-dependent glutamate transporters activates AMPA/kainate receptors to cause injury to glia and myelin. Moreover, a toxic effect played by homocysteine has been reported in primary cultures of astrocytes as a result of impaired mitochondrial metabolism [25]. In the light of these considerations, higher tHcy levels might contribute to the neonatal WMA throughout several interplayed mechanisms at various molecular and cellular levels. Notably, we could only venture the induction of oxidative stress by higher tHcy because no specific oxidative parameters were determined in our newborns. de Kremer and Grosso first reported that antepartum high tHcy levels in mothers were significantly related to HI encephalopathy in their offspring [26]. Molloy et al. (2002) showed how plasma folate levels exert the highest influence on maternal tHcy, thus suggesting that lowering of maternal tHcy might be the key factor to prevent the subsequent risks in the fetus [27]. In contrast, Maayan-Metzger et al. recently reported increased tHcy levels positively related to BW, GA, multiparity, and MTHFR C677T but not A1298C polymorphisms. No association emerged between high tHcy levels and neonatal complications including those that can be attributed to vascular etiology such as PVL, necrotizing enterocolitis, and retinopathy [28]. Similarly Kenet et al. found no association in preterm infants between perinatal complications and thrombophilia [29]. Sturtz et al. reported on a trend for lower tHcy among infants with IVH but merely attributed these findings to nonfeeding of this very sick children group [30]. The first study investigating the subsequent relationship between thrombophilic risk factors and pattern of brain injury in full term infants with NE showed that the predominant impairment of WM/WS brain regions rather than BGT regions has been related to higher tHcy levels and MTHFR 677 CT or TT genotypes [2]. However, additional acquired factors such as emergency cesarean section, hypoglycemia < 2.0 mmol/L, Apgar scores at 1 and 5 min, umbilical artery pH, and neonatal seizures played a significant causative role in this cohort [2]. Pogliani et al. showed that no significant differences were observed between the mothers, homozygous for MTHFR C677T, among their newborns with or without evidence of cUS abnormalities, with regards to age, ethnicity, type of delivery, tHcy, genetic thrombophilic factors, and drugs during pregnancy. There were no significant differences in gestational age, birth weight, umbilical cord pH, Apgar scores, or small-for-gestational age babies [31]. Abnormal cUS scans were detected in spite of maternal folic acid supplementation and antithrombotic prophylaxis during pregnancy [31]. The same authors showed a high incidence of cerebral abnormalities in neonates born to women with C677T homozygous mutation in the MTHFR gene detected by cUS [32].

The causal relationship between MTHFR A1298C polymorphisms on premature birth and neonatal WMA was less reported than MTHFR C677T variant. Moreover, studies on the role of MTHFR A1298C polymorphisms and pediatric cerebrovascular disorders gave conflicting results. MTHFR A1298C variant has been associated with ischemic stroke mainly in Asian but not in Caucasian populations [33, 34]. However, other studies did not demonstrate this correlation [35]. Two female cousins with neonatal stroke, one with compound heterozygous A1298C and C677T MTHFR variants and the other with heterozygous A1298C polymorphism, were reported [36]. An increased risk of small-for-gestational age (SGA) outcome was associated with maternal C677T and A1298C MTHFR polymorphisms in a population of sixty-six women who gave birth to one or more SGA babies [37]. Cizmeci et al. first reported a case of cerebral sinovenous thrombosis associated with MTHFR A1298C mutation in the neonatal period [38]. Conversely, homozygosity for MTHFR 1298 resulted in being protective against severe preeclampsia, fetal growth restriction, placental abruption, stillbirth, or neonatal death in a wide population of healthy nulliparous women [39]. In conclusion, both acquired (GA and perinatal events) or genetic predisposing antenatal factors were significantly associated with adverse neonatal outcome and WMA in our population of newborns, both involving activation of neurotoxicity and oxidative stress. Possibly, newborns with antenatal thrombophilic risk factors might request specific perinatal assessment, treatment, and care. Finally, the role of A1298C polymorphism may be taken into account for prenatal assessment and treatment counseling.

## Figures and Tables

**Table 1:** Demographic, clinical, biochemical, neuroimaging and frequency features of the MTHFR genotypes. All genotypes were in Hardy-Weinberg equilibrium.

Patients	*N* = 171	
Sex *N* (%)	81 M (47.4%)	
Gestational age		
≥37	126 (73.7%)	
<37	45 (26.3%)	
Type of pregnancy		
Physiologic	104/171 (60.8%)	
Pathologic	67/171 (39.2%)	
Single	157/171 (91.8%)	
Twin	14/171 (8.2%)	
Drugs' intake during gestation		
Steroid	33/171 (19.3%)	
Tocolytics	23/171 (13.5%)	
Progestins	47/171 (27.5%)	
Antiaggregant and/or anticoagulant	4/171 (2.3%)	
Antenatal complications		
Preeclampsia	5/171 (2.9%)	
PROM	3/171 (1.8%)	
Perinatal complications		
Fetal bradycardia	18/171 (10.5%)	
Flussimetry	6/171 (3.5%)	
Meconium-stained liquor	3/171 (1.7%)	
Neonatal complications		
Apgar score (1 min)	8.25 (SD ± 1.7)	
Apgar score (5 min)	9.35 (SD ± 1.1)	
Birth weight (percentile)	48.95 (SD ± 28.1)	
Perinatal infections	10/171 (5.8%)	
Neonatal seizures	5/171 (2.9%)	
Neuroimaging findings		
WMA	36/171 (21.1%)	
Ventricular Dilatation	6/36 (16.6%)	
BGT	2/171 (1.1%)	
IVH Grade I-II	13/171 (7.6%)	
Ventriculo-Peritoneal shunt catheter	6/13 (46.1%)	
tHcy *µ*M/L (mean ± SD)		
≥37 GA	9.74 (SD ± 3.98)	
<37 GA	10.55 (SD ± 4.29)	

Frequency of MTHFR C677T and A1298C genotypes (*N* = 77 pts)		

≥37 wks (*N* = 38)	MTHFR677	19CT (50%)
		4TT (10.5%)
	MTHFR1298	6AC (15.7%)
		1CC (2.6%)

<37 wks (*N* = 39)	MTHFR677	25CT (64.1%)
		4TT (10.2%)
	MTHFR1298	20AC (51.2%)
		1CC (2.5%)

GA: gestational age; tHcy: total homocysteine; MTHFR: methylenetetrahydrofolate reductase; SD: standard deviation; WMA: white matter abnormalities; BGT: basal ganglia/thalamus; IVH: intraventricular haemorrhage.

**Table 2:** *P* values of *Spearman correlation test* between plasma tHcy levels and WMA with nonparametric variables.

Significance of covariation of the examined variables and tHcy levels	*P* values
Sex (M versus F)	0.223
Type of pregnancy	0.225
Steroids	0.718
Tocolytics	0.332
Progestins	0.240
Cardioaspirin	0.084
Emergency cesarean section	0.352
Preeclampsia	0.413
PROM	**0.013** ^*^
Bradycardia	0.636
Altered flussimetry	0.664
Fetal distress	0.444
BW	0.531
GA	0.670
Apgar 1 min	0.446
Apgar 5 min	0.298
WMA	0.081
IVH	0.864
Ventricular-peritoneal catheter	0.454
PFO	**0.042** ^*^
Neonatal infections	0.201
Neonatal seizures	0.281
MTHFR genotypes	
677CT	0.276
677TT	0.053
1298AC	**0.000** ^*^
1298CC	0.232
677CT/1298AC	**0.000** ^*^

Nonparametric variables correlated to WMA	*P* values

677CT	**0.041** ^*^
677TT	0.848
1298AC	**0.000** ^*^
677CT/1298AC	**0.000** ^*^

^*^Statistical significance. tHcy: total homocysteine; MTHFR: methylenetetrahydrofolate reductase; WMA: white matter abnormalities; IVH: intraventricular hemorrhage; PROM: premature rupture of membranes; BW: birth weight; PFO: patent foramen ovale; CT and TT: heterozygous and homozygous MTHFR 677 genotypes; AC and CC: heterozygous and homozygous MTHFR 1298 genotypes.

**Table 3 tab3:** Linear regression model showed dependence of Apgar score at 5 minutes, PFO, and PROM from tHcy levels.

tHcy	*P* values
Type of pregnancy	0.958
BW	0.624
GA	0.374
Apgar 1	0.065
Apgar 5	**0.002** ^*^
Steroids	0.066
Tocolytics	0.545
Progestins	0.535
Cardioaspirin	0.167
Preeclampsia	0.447
Emergency cesarean section	0.704
PROM	**0.007** ^*^
Bradycardia	0.945
PFO	**0.024** ^*^
Altered flussimetry	0.469

^*^Statistical significance, *P* < 0.05. GA: gestational age; tHcy: total homocysteine; MTHFR: methylenetetrahydrofolate reductase; PROM: premature rupture of membranes; BW: birth weight; PFO: patent foramen ovale.

**Table 4:** Linear regression model showed dependence of WMA from tHcy levels, GA, Apgar scores at 1 and 5 minutes, 1298AC, and 677CT/1298AC MTHFR genotypes. IVH resulted in being dependent on GA and bradycardia by the same model.

WMA	*P* values
tHcy	**0.014** ^*^
GA	**0.000** ^*^
Apgar 1	**0.000** ^*^
Apgar 5	**0.000** ^*^
Altered flussimetry	0.999
Preeclampsia	0.999
PROM	0.096
677CT	0.297
677TT	0.065
1298AC	**0.000** ^*^
1298CC	0.270
677CT/1298AC	**0.000** ^*^
IVH	
tHcy	0.862
GA	**0.000** ^*^
Preeclampsia	0.314
PROM	0.138
Altered flussimetry	0.410
Bradycardia	**0.000** ^*^
677CT	0.726
677TT	0.522
1298AC	0.101
1298CC	0.999
677CT/1298AC	0.390

^*^Statistical significance. *P* < 0.05. tHcy: total homocysteine; MTHFR: methylenetetrahydrofolate reductase; WMA: white matter abnormalities; IVH: intraventricular hemorrhage; PROM: premature rupture of membranes; BW: birth weight; GA: gestational age; PFO: patent foramen ovale; CT and TT: heterozygous and homozygous MTHFR 677 genotypes; AC and CC: heterozygous and homozygous MTHFR 1298 genotypes.
